# Nature Exposure and Problematic Smartphone Use Among Chinese High School Students: The Mediating Roles of Anxiety and Self-Control

**DOI:** 10.3390/bs16061019

**Published:** 2026-06-18

**Authors:** Li Wu, Ting Han, Gengfeng Niu, Xiaxia Xu

**Affiliations:** 1 School of International Equestrianism, Wuhan Business University, Wuhan 430056, China; 2 Research Center for Modern Equine Industry Development, Wuhan 430056, China; 3 Key Laboratory of Adolescent Cyberpsychology and Behavior (CCNU), Ministry of Education, Wuhan 430079, China; 4 College of Urban and Environmental Sciences, Central China Normal University, Wuhan 430079, China; 5 Key Laboratory of Human Development and Mental Health of Hubei Province, School of Psychology, Central China Normal University, Wuhan 430079, China; 6 Center for Research on Internet Literacy and Behavior, Central China Normal University, Wuhan 430079, China

**Keywords:** nature exposure, problematic smartphone use, anxiety, self-control, adolescents

## Abstract

Problematic smartphone use (PSU) has become an increasingly important public health concern among adolescents, yet the potential protective role of restorative environmental experiences (nature exposure) remains insufficiently understood. Under the perspective of Stress Reduction Theory (SRT) and Attention Restoration Theory (ART), this cross-sectional study examined the association between nature exposure and adolescent PSU, with anxiety and self-control tested as potential mediators. The sample comprised 700 high school students recruited from several high schools in Qinghai Province, China (52.00% female; *M* age = 17.01 years, *SD* = 0.78). Nature exposure, anxiety, self-control, and PSU were assessed using self-report measures. The results showed that nature exposure was negatively associated with PSU; anxiety and self-control significantly mediated this association both independently and sequentially. Specifically, more nature exposure was associated with lower anxiety and higher self-control, which, in turn, were associated with lower PSU. These findings suggest that restorative environmental experiences may be associated with reduced vulnerability to PSU through interconnected affective and self-regulatory processes. The present study extends existing literature by integrating emotional and attentional restoration perspectives within a unified framework linking nature exposure to adolescent PSU, and provides implications for the prevention and intervention of PSU.

## 1. Introduction

Problematic smartphone use (PSU) refers to uncontrolled and excessive smartphone use, which leads to significant impairments to individuals’ physiological, psychological, and social functioning (Billieux et al., 2015; García-Canalejas et al., 2025; Y. Li et al., 2020). PSU is characterized by compulsive smartphone engagement and diminished behavioral control despite negative consequences (Elhai et al., 2017; Sapacz et al., 2016; Shi et al., 2025). Adolescence may represent a particularly vulnerable developmental period for PSU due to heightened emotional reactivity and still-developing regulatory control systems (Extremera et al., 2019; Silvers et al., 2012).

Moreover, national epidemiological evidence indicates that behavioral and emotional problems among children and adolescents have increased substantially over the past 30 years, highlighting adolescence as a critical developmental stage for investigating risk and protective factors related to PSU (Cui et al., 2021). Recent evidence indicates that the prevalence of PSU has increased substantially, with a meta-analysis reporting a pooled prevalence of 27.0%, particularly among adolescents (Meng et al., 2022). A meta-analysis also revealed that PSU is associated not only with lowered subjective well-being with a moderate effect (Chen et al., 2025), but also with a range of mental health problems, including depression, anxiety, and eating disorders (Malek Mohammadi et al., 2024; J. Wang et al., 2023). Given its high prevalence and detrimental psychological consequences among adolescents, adolescents’ PSU has become an important public health concern throughout the world.

Therefore, further research is needed to clarify the underlying psychological mechanisms and potential influencing factors (especially the protective factors) associated with adolescent PSU.

### 1.1. Nature Exposure and PSU

Given the high prevalence and adverse consequences of PSU among adolescents, increasing attention has been paid to identifying the protective factors that may mitigate the risk of PSU. Among these factors, nature exposure has emerged as a potentially important environmental resource for promoting emotional well-being and reducing stress (Bratman et al., 2021). Nature exposure generally refers to individuals’ contact with natural environments, including green spaces, vegetation, parks, and wilderness settings (Holland et al., 2021). From this perspective, natural environments and smartphone-based digital environments may exert opposing influences on emotional and self-regulatory functioning, with the former facilitating psychological restoration and the latter contributing to sustained cognitive and emotional demands (Kaplan & Berman, 2010; Ulrich et al., 1991). The pervasive use of smartphones may continuously expose individuals to reward-related cues and social feedback, thereby sustaining attentional capture and reducing opportunities for psychological recovery (Brand et al., 2019; Meshi et al., 2015). From a technology engagement perspective, smartphone use is characterized by continuous intermittent reinforcement schedules, which may strengthen habitual checking behaviors and reduce opportunities for cognitive disengagement (Oulasvirta et al., 2012). Against this background, theoretical perspectives have been proposed to explain the potential restorative role of nature exposure.

A growing body of research has demonstrated that nature exposure exerts beneficial effects on individuals’ physical, psychological, and cognitive functioning. According to the Stress Reduction Theory (SRT), exposure to natural environments facilitates emotional recovery and reduces psychological stress and anxiety (Ulrich, 2023). In addition, the Attention Restoration Theory (ART) also proposes that natural environments help restore depleted attentional and self-regulatory resources, thereby enhancing individuals’ self-control capacity (Kaplan, 1995; Kaplan & Berman, 2010). Furthermore, the Conservation of Resources (COR) theory also posits that sufficient resources enable individuals to cope with stress and maintain self-regulatory capacity, thereby reducing reliance on maladaptive coping strategies such as problematic smartphone use (Hobfoll, 1989).

Together, these theoretical perspectives suggest that nature exposure may reduce technology-related behaviors through affective and self-regulatory restoration processes. Consistent with these theoretical perspectives, accumulating empirical evidence suggests that greater nature exposure is associated with lower levels of PSU and other addictive behaviors. Studies, for example, have shown that individuals with greater nature exposure tend to report lower problematic smartphone use (Minor et al., 2023; Zhu, 2024). In addition, research has demonstrated a negative association between nature connectedness and internet addiction (B. Wang et al., 2024). Collectively, these findings suggest that nature exposure may buffer against PSU by restoring depleted affective and self-regulatory resources in digitally saturated environments. Building on these theoretical and empirical insights, the present study proposes that nature exposure is negatively associated with PSU. Accordingly, this study proposes the following hypothesis:

**H1.**  *Nature exposure is negatively associated with PSU.*

### 1.2. The Mediating Role of Anxiety and Self-Control

This study further advances existing literature by proposing an integrated restorative framework in which nature exposure may influence PSU through both affective and self-regulatory restoration processes. Although previous studies have shown that nature exposure is associated with lower anxiety and enhanced self-control, and that both factors are related to PSU, these affective and self-regulatory mechanisms have largely been examined separately. Consequently, it remains unclear whether affective restoration and self-regulatory restoration operate as independent pathways or as interconnected processes linking nature exposure to PSU.

### 1.3. The Mediating Role of Anxiety

Drawing on SRT, exposure to natural environments is assumed to facilitate emotional recovery by reducing physiological and psychological stress responses, including anxiety (Joye & Dewitte, 2018; Ulrich et al., 1991). Neurophysiological research also supports this perspective, which has shown that exposure to nature may reduce activation in stress-related brain regions such as the amygdala (Sudimac et al., 2022). Consistent with this framework, relevant empirical studies also have demonstrated that both real-world and virtual nature exposure can effectively reduce anxiety levels (Lopes & Falk, 2024; Zhang et al., 2023).

At the same time, anxiety has been consistently identified as a significant risk factor for problematic smartphone use and PSU. Meta-analytic and empirical evidence indicates that individuals with higher levels of anxiety are more likely to engage in excessive smartphone use (de-Sola et al., 2026; Dou et al., 2024; Elhai et al., 2017). This relationship can be explained by compensatory and emotion regulation frameworks, which propose that smartphones provide readily accessible distraction, social reassurance, and emotional relief, leading anxious individuals to rely on excessive smartphone use as a maladaptive emotion regulation and compensatory coping strategy (Shahidin et al., 2022).

Taken together, these theoretical and empirical perspectives suggest that anxiety may function as a critical affective mechanism linking nature exposure to PSU. Specifically, nature exposure may reduce individuals’ anxiety levels, which, in turn, decreases their reliance on smartphones for emotional compensation and maladaptive coping. Consistent with this perspective, prior research has shown that nature exposure may protect against problematic behaviors, such as problematic alcohol use, through reducing negative affective experiences (e.g., distress and shame) (Almog et al., 2022), and that negative emotions (e.g., depressive symptoms) may mediate the link between adverse social environments and video game addiction (K. Li et al., 2024). These findings collectively provide direct evidence that negative emotions may serve as important mediating mechanisms linking environmental factors to addictive behaviors. In the present study, negative emotions (such as anxiety) are proposed as a particularly relevant affective pathway underlying the association between nature exposure and PSU. Accordingly, this study proposes the following hypothesis:

**H2.**  *Anxiety mediates the relationship between nature exposure and PSU.*

### 1.4. The Mediating Role of Self-Control

Self-control refers to a dynamic self-regulatory system that enables individuals to inhibit impulses and prioritize long-term goals over immediate gratification, with attentional regulation being a central mechanism (Duckworth et al., 2016; Englert et al., 2015; Werner & Ford, 2023). According to ART, prolonged cognitive demands would deplete directed attentional resources supported by cognitive control system, whereas exposure to natural environments facilitates attentional recovery (Kaplan, 1995). Because self-control relies heavily on directed attention resources, nature exposure may enhance individuals’ self-control capacity through attentional restoration processes (Kaplan & Berman, 2010). Consistent with this perspective, neurophysiological studies have shown that nature exposure may improve inhibitory attentional processing and modulate neural mechanisms underlying cognitive control (Bell et al., 2026).

In the context of smartphone use, individuals with lower self-control may experience greater difficulty resisting the immediate rewards provided by smartphones, thereby increasing the likelihood of habitual and compulsive use behaviors. A large body of cross-sectional and longitudinal empirical evidence has consistently demonstrated a significant negative association between self-control and problematic smartphone use as well as PSU among adolescents, and self-control has been identified as an important protective factor against addictive smartphone behaviors in adolescent populations (Han et al., 2017; Q. Wang et al., 2024; Zhao et al., 2024).

In summary, these findings provide converging evidence that self-control may mediate the relationship between nature exposure and PSU. Specifically, exposure to natural environments may enhance individuals’ self-control capacity through attentional restoration, thereby reducing excessive and maladaptive smartphone use behaviors. Consistently, prior research has indicated that environment-related factors may shape problematic behaviors through self-control processes. For example, supportive family environments characterized by cohesion, expressiveness, and low conflict have been shown to reduce problematic internet use indirectly via enhanced self-control (Safari et al., 2025), and parental control has similarly been found to exert indirect effects on adolescents’ problematic internet use via self-control (X. Li et al., 2013). Taken together, these findings converge to highlight self-control as a key psychological pathway linking environmental experiences to addictive online behaviors. Accordingly, this study proposes the following hypothesis:

**H3.**  *Self-control mediates the relationship between nature exposure and PSU.*

### 1.5. The Serial Mediation of Anxiety and Self-Control

Anxiety and self-control may operate in a sequential manner linking nature exposure to PSU. Prior research suggests that heightened anxiety can impair self-regulatory functioning by consuming directed attentional and self-regulatory resources (Heatherton & Wagner, 2011; Hofmann et al., 2012). When individuals experience anxiety, increased emotional processing may reduce the resources available for behavioral regulation, which may weaken self-control capacity. Reduced self-control, in turn, has been consistently associated with greater risk of problematic smartphone use among adolescents (Q. Wang et al., 2024; Zhao et al., 2024). Accordingly, anxiety may indirectly contribute to PSU by undermining self-control. This sequential pathway reflects the dynamic interplay between affective restoration and self-regulatory restoration, whereby reductions in anxiety preserve regulatory resources necessary for effective self-control (Heatherton & Wagner, 2011; Hofmann et al., 2012). In this sense, affective restoration may serve as a prerequisite for the recovery of self-regulatory functioning, thereby decreasing vulnerability to maladaptive smartphone use behaviors (Kaplan & Berman, 2010).

Integrating these findings, nature exposure may reduce PSU through a sequential pathway in which reduced anxiety is associated with improved self-control, ultimately decreasing PSU. Accordingly, we hypothesize that:

**H4.**  *Anxiety and self-control sequentially mediate the relationship between nature exposure and PSU.*

### 1.6. The Current Study

Building on SRT, ART, the present study constructed a serial mediation model linking nature exposure to PSU through anxiety and self-control among adolescents. Adolescence represents a critical developmental period during which heightened affective reactivity and ongoing maturation of executive control systems may amplify the environmental restoration processes (Casey et al., 2008; Steinberg, 2008). Specifically, this study addresses the fragmented nature of existing research by integrating affective restoration and self-regulatory restoration processes within a unified sequential framework. By framing nature exposure as a restorative environmental resource, the present study extends existing PSU research beyond individual-level psychological risk factors and highlights the potential role of environmental restoration processes in reducing maladaptive technology-related behaviors. Whereas prior studies have typically examined emotional factors (e.g., anxiety) and self-regulatory factors (e.g., self-control) in isolation (Kwak et al., 2022), the potential sequential interplay between affective restoration and self-regulatory restoration remains insufficiently understood.

**Figure 1:**
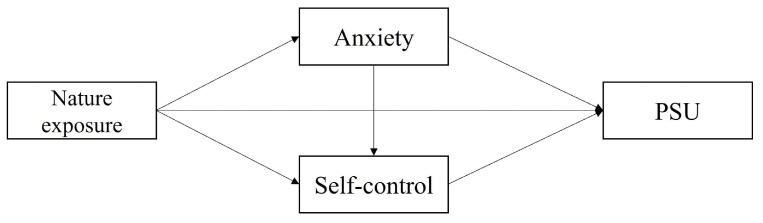
Hypothesized model.

This study proposes a sequential association in which greater nature exposure is associated with lower anxiety, lower anxiety is associated with higher self-control, and higher self-control is associated with lower PSU. By integrating affective and self-regulatory perspectives, this study aims to provide a more comprehensive understanding of how nature exposure is associated with lower levels of PSU. Specially, the present study aimed to examine the direct and indirect effects of nature exposure on PSU via anxiety and self-control in a serial mediation model (see Figure 1).

## 2. Methods

### 2.1. Participants and Procedure

A total of 713 high school students from several public schools in Qinghai Province, China, were initially recruited to complete the survey. Two participants who failed to answer more than half of the items and 11 participants who did not pass the attention check items were excluded; the final analytic sample comprised 700 participants (98.18%), including 364 females (52.00%) and 336 males (48.00%), with a mean age of 17.01 years (*SD* = 0.78).

All participants were recruited from several high schools through convenience sampling. Prior to the survey, parental/guardian consent was obtained in advance through the assistance of school teachers, and student assent was collected on the day of data collection. Both students and their guardians were informed that participation was entirely voluntary and that students could withdraw at any time without any penalty. Questionnaires were administered collectively in study rooms to avoid interfering with regular academic classes. Teachers organized the administration and ensured students’ safety; however, they were not involved in or able to view students’ responses, and care was taken to minimize any potential influence on participants’ answers. As an incentive for participation, each student received a pen. All questionnaires were collected anonymously to ensure confidentiality. This study was conducted in accordance with the Declaration of Helsinki, and approved by the Ethics Committee of Central China Normal University. Informed consent was obtained from all subjects involved in this study.

### 2.2. Measurements

#### 2.2.1. Problematic Smartphone Use

The 17-item Chinese version of the Mobile Phone Addiction Index (MPAI; H. Huang et al., 2014) was used to assess PSU across four subscales: Inability to Control Cravings Subscale (ICCS), Feeling Anxious and Lost Subscale (FALS), Withdrawal and Escape Subscale (WES), and Productivity Loss Subscale (PLS). Participants rated each item on a 5-point Likert scale ranging from 1 (*never*) to 5 (*always*). Scale scores were calculated by averaging item responses, with higher scores indicating greater levels of PSU. The Chinese version of the MPAI has demonstrated good internal consistency in Chinese adolescents (Cronbach’s *α* = 0.93; Cheng et al., 2024). In the present study, the scale also showed satisfactory reliability, with a Cronbach’s *α* of 0.92.

#### 2.2.2. Nature Exposure

Nature exposure was measured using the 4-item Nature Exposure Scale (NES; Kamitsis & Francis, 2013), which assesses individuals’ levels of exposure to nature in everyday life and activities as well as exposure outside of everyday environments. Items are rated on a 5-point Likert scale ranging from 1 (*low/not much*) to 5 (*high/a great deal*). Scale scores were calculated by averaging item responses, with higher scores indicating greater levels of exposure to nature. In the present study, the translation and back-translation process was conducted by bilingual researchers in psychology who are fluent in both Mandarin and English. The results of the confirmatory factor analysis (CFA) indicated satisfactory structural validity (*χ*^2^ = 27.95, *df* = 6, *χ*^2^/*df* = 4.66, RMSEA = 0.05, CFI = 0.93, NFI = 0.92, IFI = 0.92). Cronbach’s *α* coefficient in the current study was 0.89.

#### 2.2.3. Anxiety

The 7-item Generalized Anxiety Disorder Scale (GAD-7; Spitzer et al., 2006) was used to assess generalized anxiety symptoms experienced over the past two weeks. Items are rated on a 4-point Likert scale ranging from 0 (*not at all*) to 3 (*nearly every day*). Scale scores were calculated by averaging item responses, with higher total scores indicating greater severity of generalized anxiety symptoms. The Chinese version of the GAD-7 has demonstrated good reliability (Cronbach’s *α* = 0.93–0.95) and validity among Chinese adolescents aged 10 to 17 years (J. Sun et al., 2021). In the present study, the scale also showed excellent internal consistency, with a Cronbach’s *α* coefficient of 0.92.

#### 2.2.4. Self-Control

The 19-item Chinese version of the Self-Control Scale (SCS; Tan & Guo, 2008) comprises five subscales: Impulse Control, Healthy Habits, Resisting Temptation, Work Ethic, and Entertainment Restraint. Items are rated on a 5-point Likert scale ranging from 1 (*not at all like me*) to 5 (*very much like me*). Scale scores were calculated by averaging item responses, with higher scores indicating higher levels of self-control. The Chinese version of the SCS has demonstrated good reliability in Chinese adolescent samples (Cronbach’s *α* = 0.84; Liu et al., 2016). In the present study, the scale showed excellent internal consistency, with a Cronbach’s *α* of 0.93.

### 2.3. Data Analysis

The data analysis proceeded in sequential phases. First, common method variance was assessed using a comparison of a single-factor CFA model with the hypothesized four-factor measurement model. Then, descriptive statistics were calculated to examine the distributional characteristics of the study variables, including means and standard deviations. Subsequently, Pearson’s correlation analyses were performed to assess the associations among the variables. Finally, Model 6 of the SPSS PROCESS macro (Version 4.1) was used to test the serial mediating roles of anxiety and self-control in the relationship between nature exposure and PSU. The significance of indirect effects was evaluated using bias-corrected bootstrap confidence intervals based on 5000 resamples. Indirect effects were considered significant when the 95% confidence interval did not include zero. Statistical conclusions were based on both statistical significance (two-tailed, *p* < 0.05) and effect size estimates. For correlation coefficients, small, medium, and large effects were defined as *r* = 0.10, 0.30, and 0.50, respectively (Cohen, 1992). All analyses were conducted using SPSS 27.0 and AMOS 26.0.

## 3. Results

### 3.1. Common Method Bias Test

To test the potential common method variance, a single-factor confirmatory factor analysis (CFA) model was compared with the hypothesized four-factor measurement model (Iverson & Maguire, 2000). The results showed that the four-factor model provided a substantially better fit to the data than the single-factor model. Specifically, the differences in fit indices were ΔCFI = 0.184, ΔTLI = 0.192, and ΔRMSEA = 0.025. This indicates that there exists no significant common method bias in this study.

### 3.2. Descriptive Statistics and Correlation Analysis

Descriptive statistics and Pearson’s correlation coefficients for all study variables are presented in Table 1. Nature exposure was significantly negatively correlated with PSU (*r* = −0.53, *p* < 0.001) and anxiety (*r* = −0.41, *p* < 0.001), and significantly positively correlated with self-control (*r* = 0.50, *p* < 0.001). PSU was positively correlated with anxiety (*r* = 0.44, *p* < 0.001) and negatively correlated with self-control (*r* = −0.67, *p* < 0.001). In addition, anxiety was significantly negatively correlated with self-control (*r* = −0.41, *p* < 0.001).

### 3.3. Analysis of Serial Mediation Model

Prior to conducting the mediation analyses, the assumptions underlying the regression models were examined. Specifically, regression models with Anxiety, Self-control, and PSU as the dependent variables were assessed for residual independence, multicollinearity, normality of residuals, homoscedasticity, linearity, and influential cases. The results indicated that all assumptions were adequately satisfied across the three regression models, supporting the appropriateness of the subsequent mediation analyses.

A serial mediation analysis was conducted using Model 6 of the PROCESS macro for SPSS 27.0 to examine whether anxiety and self-control mediated the association between nature exposure and PSU. As the established relevance of gender differences in the main variables (Farhane-Medina et al., 2022; Y. Huang et al., 2026), gender was included as a control variable. The indirect effects were tested using a bias-corrected bootstrap procedure with 5000 resamples and 95% confidence intervals. The regression coefficients are presented in Table 2.

The mediation analysis examined the total, direct, and indirect effects of nature exposure on PSU. The total effect was statistically significant (effect = −0.44, *SE* = 0.03, 95% CI [−0.50, −0.38]). After controlling for the mediators, the direct effect remained significant (effect = −0.18, *SE* = 0.03, 95% CI [−0.24, −0.12]), accounting for 40.64% of the total effect.

**Figure 2:**
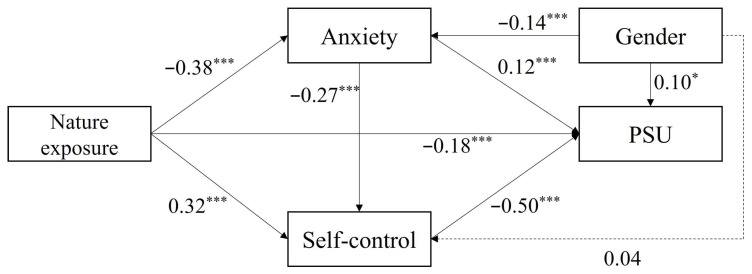
Serial Mediation Model Linking Nature Exposure to PSU through Anxiety and Self-Control; * *p* < 0.05, *** *p* < 0.001.

Three statistically significant indirect pathways were identified. The indirect effect through anxiety was significant (effect = −0.05, *SE* = 0.01, 95% CI [−0.08, −0.02]), accounting for 10.50% of the total effect. The indirect effect via self-control was also significant (effect = −0.16, *SE* = 0.02, 95% CI [−0.20, −0.12]), accounting for 36.76% of the total effect. In addition, the serial indirect effect through anxiety and self-control was significant (effect = −0.05, *SE* = 0.01, 95% CI [−0.07, −0.03]), accounting for 11.87% of the total effect. These percentages are provided for descriptive purposes only and should not be interpreted causally. Collectively, these results indicate that anxiety and self-control statistically mediated the association between nature exposure and PSU through both parallel and serial pathways. The results are showed in Table 3 and Figure 2.

## 4. Discussion

This study examined the association between nature exposure and general PSU, and tested anxiety and self-control as independent and serial mediators in a non-clinical adolescent sample. The results showed a significant negative association between nature exposure and PSU. Moreover, both anxiety and self-control served as significant independent mediators, and a significant serial mediation pathway from anxiety to self-control was identified after controlling gender. These findings provide initial evidence for a dual-pathway pattern of statistical associations between nature exposure and PSU via affective and self-regulatory processes.

### 4.1. Association Between Nature Exposure and PSU

The present findings indicated that nature exposure was negatively associated with PSU, supporting H1 and consistent with prior evidence showing that greater engagement with natural environments is linked to lower problematic smartphone use and other maladaptive digital behaviors (Minor et al., 2023; Zhu, 2024). Importantly, the current findings extend previous research by suggesting that restorative environmental experiences may represent a protective contextual factor against adolescent PSU. This association can be explained through complementary theoretical perspectives.

From the perspectives of SRT and ART, the negative association between nature exposure and problematic smartphone use may be explained by the resource-replenishing effects of natural environments. Specifically, exposure to natural settings helps alleviate physiological and psychological stress, thereby reducing negative affect and anxiety, while also restoring depleted directed attention and enhancing executive functioning (Kaplan, 1995; Kaplan & Berman, 2010; Ulrich, 2023). Together, these restorative processes may replenish the emotional and cognitive resources required for adaptive self-regulation. Building on the restorative perspective, the present findings can also be interpreted from the perspective of COR theory as a complementary framework, which suggests that individuals with more sufficient emotional and cognitive resources are better able to cope with daily stressors and maintain self-regulation, thereby reducing reliance on maladaptive coping behaviors such as excessive smartphone use (Hobfoll, 1989). Moreover, consistent with the gain spiral principle, initial resource restoration may facilitate subsequent resource acquisition, thereby reinforcing adaptive functioning and producing a sustained reduction in problematic smartphone use (Hobfoll, 2001).

### 4.2. The Mediating Effect of Anxiety

The present findings supported H2, indicating that anxiety served as a key affective mechanism mediating the association between nature exposure and PSU. This result is consistent with previous research showing that exposure to natural environments is associated with emotional benefits, whereas general and social anxiety are important risk factors for PSU (Bratman et al., 2021; Vitale & Bonaiuto, 2024; Y. Li et al., 2020; Ran et al., 2022).

This finding may be understood within the SRT, which proposes that exposure to natural environments facilitates emotional recovery and reduces anxiety-related stress responses (Ulrich et al., 1991; Vitale & Bonaiuto, 2024). In turn, lower anxiety may reduce adolescents’ reliance on smartphones as a maladaptive coping strategy for emotional compensation. In addition, the Compensatory Internet Use Theory also stated that, individuals experiencing elevated anxiety are more likely to engage in excessive smartphone use to escape from or alleviate psychological distress. By contrast, adolescents with lower anxiety may have less need to seek emotional relief through smartphone use (Kardefelt-Winther, 2014). Thus, nature exposure may be associated with lower vulnerability to PSU through anxiety-related emotional restoration. Importantly, the present study extends restorative environment research by suggesting that affective restoration processes may also be relevant to maladaptive technology-related behaviors in digital contexts.

### 4.3. The Mediating Effect of Self-Control

The present findings supported H3, indicating that self-control mediated the association between nature exposure and PSU. These findings suggest that self-regulatory functioning may represent an important psychological pathway linking restorative environmental experiences to reduced vulnerability to problematic smartphone use among adolescents. Previous studies have consistently identified self-control as a key mediator linking both environmental and individual factors (e.g., parenting, loneliness, and future time perspective) to PSU (X. Li et al., 2021; Q. Li et al., 2026; Pan et al., 2023; Peng et al., 2022). The current study extends this line of research by demonstrating that self-control may also operate within the psychological process linking nature exposure to PSU.

Adolescents with lower self-control may experience greater difficulty resisting immediate rewards and habitual checking behaviors associated with smartphone use; so, they may engage in smartphone use more frequently for social, entertainment, and reward-seeking purposes, all of which may provide immediate gratification and increase vulnerability to PSU (Jiang & Zhao, 2016). This interpretation is also consistent with meta-analytic evidence demonstrating a relatively strong negative association between self-control and PSU (Ding et al., 2022). More importantly, relevant studies show that nature exposure improves self-regulation in mentally fatigued populations (e.g., H. Sun et al., 2024), the present findings further suggest that attentional restoration may extend to the domain of PSU. This interpretation aligns with ART, which proposes that natural environments replenish directed attentional resources following sustained cognitive demands. In particular, restorative environments may facilitate executive attentional processes such as attentional control, working memory, and cognitive flexibility, all of which are essential for effective self-regulation (Stevenson et al., 2018). Extending this perspective, the present study suggests that restored attentional capacity may strengthen self-control, thereby reducing vulnerability to PSU.

### 4.4. The Serial Mediating Effect of Anxiety and Self-Control

A notable finding of the present study was that anxiety and self-control formed a sequential pathway linking nature exposure to PSU, thereby supporting H4. The findings suggest that affective and self-control processes may be interconnected, consistent with Ye et al. (2025), in the association between restorative environmental experiences and adolescent PSU.

Persistent anxious arousal may impair attention control by reducing the efficiency of executive processes required for effective behavioral regulation, thereby making it more difficult for individuals to maintain inhibitory control over excessive smartphone use (Eysenck et al., 2023). In line with this perspective, previous research also has shown that individuals with higher levels of social anxiety are more vulnerable to self-control depletion following social interactions, suggesting that anxiety-related processes may directly undermine regulatory capacity (Blackhart et al., 2015). Importantly, the present findings provide support for an integrated restorative perspective combining SRT and ART. Whereas SRT primarily emphasizes emotional restoration through reductions in stress and anxiety (Yao et al., 2021), ART focuses on the recovery of directed attentional resources and the alleviation of attentional fatigue (Joye & Dewitte, 2018). The current sequential mediation findings suggest that these restorative processes may operate in a cascading manner, such that reductions in negative affect facilitate subsequent restoration of attentional resources and self-regulatory capacity. This integrative pathway extends previous research by demonstrating how emotional and cognitive restoration processes may jointly contribute to lower vulnerability to adolescent PSU.

### 4.5. Implications

Theoretically, the present study extends existing literature by integrating ART and SRT to explain the association between nature exposure and PSU. The findings suggest that nature exposure may reduce maladaptive smartphone use through interconnected affective and self-regulatory processes, highlighting the joint role of emotional restoration and self-regulatory resource recovery in adolescents’ behavioral functioning.

The present findings also suggest several tentative practical implications. First, schools and families could integrate nature-based activities into daily routines through campus greening, outdoor physical education, and ecological or experiential learning programs, thereby providing adolescents with regular opportunities for exposure to restorative environments (Ly & Vella-Brodrick, 2024); virtual nature experiences or exposure to natural imagery may also serve as alternative means of facilitating emotional restoration and reducing stress-related arousal (Lopes & Falk, 2024). Second, given the mediating role of anxiety, interventions targeting emotional regulation may also be beneficial, and anxiety management training, mindfulness-based approaches, and stress reduction programs may help alleviate adolescents’ emotional distress; school-based prevention programs can also effectively reduce adolescents’ internalizing symptoms (Feiss et al., 2019). Third, regarding the self-control, beyond traditional effortful self-control training, self-regulatory capacity may also be supported through effortless processes, such as nature exposure, flow-like experiences, and other low-effort restorative practices (Tang et al., 2022). All these measures could be adopted to improve adolescents’ adaptation and be incorporated into prevention programs targeting PSU.

### 4.6. Limitations and Future Directions

Several limitations of the present study should be acknowledged. First, the cross-sectional design limits causal inferences, though our model was grounded in theoretical and empirical evidences, alternative explanations involving reverse causality remain plausible. Future longitudinal and experimental studies are needed to compare competing models, examine the directionality of these relationships, and provide stronger evidence for the proposed mediation pathways. Second, all variables were assessed using self-report measures, which may introduce potential biases such as social desirability and common method variance. Multi-method assessments, such as behavioral tasks, physiological indicators, ecological assessments, or digital trace data, could be adopted to improve measurement validity and enhance the ecological validity of the findings. Third, the present study employed a convenience sampling strategy and the sample was limited to Chinese high school students. These factors may introduce potential selection bias and restrict the generalizability of the findings. Future studies are encouraged to use probability sampling methods and include more diverse samples to improve the external validity. Fourth, we acknowledge as a limitation that only gender was included as a covariate. Other factors (e.g., socioeconomic status, academic pressure) may influence the observed associations, and future studies should consider incorporating a broader range of covariates to further improve the scientificity and rigor. Fifth, nature exposure was measured using a brief four-item self-report scale. Although this scale assessed general exposure to nature across different contexts, it may not fully capture the construct’s multidimensional nature (e.g., duration, frequency, quality, accessibility, biodiversity, and type of engagement). Future research should adopt more comprehensive and multidimensional assessments. Sixth, although self-control and PSU are theoretically distinct constructs, some degree of conceptual or item-level overlap may exist, particularly in aspects related to behavioral regulation and difficulties controlling smartphone use. Therefore, the observed association should be interpreted with caution, and future research should further examine the distinctiveness of these constructs using alternative measures and assessment methods. Finally, although the present study focused on anxiety and self-control as key mediators, PSU is a complex behavior influenced by multiple psychological and contextual factors. The remaining direct effect may reflect additional potential mediating pathways, such as empathy, which has been identified as a factor closely linked to both nature exposure and PSU in prior research (Busch & McCarthy, 2021; Y. Li et al., 2025). Future research may benefit from incorporating additional mediators and potential moderators to further refine the theoretical model.

## 5. Conclusions

The present study demonstrates that nature exposure is negatively associated with PSU in adolescents and identifies a sequential mediating pattern linking anxiety and self-control in this relationship. Specifically, anxiety and self-control jointly mediated the association, suggesting that restorative environmental experiences may be related to reduced problematic smartphone use through interconnected affective and self-regulatory processes.

Integrating SRT and ART, the findings are consistent with a cascading restorative framework in which lower levels of negative affective arousal are associated with higher self-regulatory capacity. This pattern may help explain individual differences in vulnerability to reward-driven and habitual smartphone use in daily digital environments.

Overall, the present study provides correlational evidence for a cascading pathway linking nature exposure to reduced PSU via anxiety and self-control. These findings extend existing literature by highlighting environmental restoration as a potential contextual factor in adolescent behavioral regulation. Future longitudinal and experimental research is needed to further validate these associations.

## Figures and Tables

**Table 1:** Descriptive Statistics and Variable Correlations (*n* = 700).

Variables	*M*	*SD*	1	2	3	4
1. Nature exposure	3.45	0.70	1			
2. PSU	3.47	0.58	−0.53 ***	1		
3. Anxiety	2.31	0.62	−0.41 ***	0.44 ***	1	
4. Self-control	3.28	0.61	0.50 ***	−0.67 ***	−0.41 ***	1

Note. *** *p* < 0.001.

**Table 2:** Test for the Standardized Regression Coefficients of the Serial Mediation Model.

Outcome Variable	Predictor Variable	*R*	*R* ^2^	*F*	*β*	*t*
PSU	Nature exposure	0.54	0.29	67.03 ***	−0.44	−13.72 ***
Anxiety	Nature exposure	0.43	0.19	38.07 ***	−0.38	−10.41 ***
Self-control	Nature exposure	0.58	0.33	61.09 ***	0.32	8.96 ***
	Anxiety				−0.27	−6.81 ***
PSU	Nature exposure	0.73	0.54	114.03 ***	−0.18	−5.81 ***
	Anxiety				0.12	3.67 ***
	Self-control				−0.50	−14.03 ***

Note. *** *p* < 0.001.

**Table 3:** Standardized Total, Direct, and Indirect Effects of the Serial Mediation Model.

	Effect	*SE*	Boot 95% CI Lower	Boot 95% CI Upper	Effect Proportion
Total effect	−0.44	0.03	−0.50	−0.38	
Direct effect	−0.18	0.03	−0.24	−0.12	40.64%
Indirect effect 1	−0.05	0.01	−0.08	−0.02	10.50%
Indirect effect 2	−0.16	0.02	−0.20	−0.12	36.76%
Indirect effect 3	−0.05	0.01	−0.07	−0.03	11.87%

Note. Indirect effect 1 = Nature exposure → Anxiety → PSU; Indirect effect 2 = Nature exposure → Self-control → PSU; Indirect effect 3 = Nature exposure → Anxiety → Self-control → PSU.

## Data Availability

The data presented in this study are available on request from the corresponding author due to privacy and ethical restrictions.
